# Preventable, Yet Unprevented: The Cervical Cancer Screening Landscape in Eastern Europe and Bulgaria

**DOI:** 10.3390/healthcare14182973

**Published:** 2026-09-11

**Authors:** Angel Yordanov, Tsvetan Yordanov, Eva Tsoneva, Ivaylo Petrov, Ihsan Hasan, Dimo Manov, Stoyan Kostov, Assia Konsoulova

**Affiliations:** 1 Department of Gynaecological Oncology, Medical University Pleven, 5800 Pleven, Bulgaria; 2 Medical Oncology Clinic, University Specialized Hospital for Active Treatment in Oncology “Prof. Ivan Chernozemski”, 1756 Sofia, Bulgaria; 3 Gynecology Clinic, Military Medical Academy, 1606 Sofia, Bulgaria; 4 Bulgarian Joint Cancer Network, 9000 Varna, Bulgaria; ivaylo.petrov@shemhahealth.com (I.P.);; 5 Department of Gynaecology and Pelvic Surgery, Hospital (MHAT), 1407 Sofia, Bulgaria; ihsan_hasanov@abv.bg; 6 Nadezhda Hospital, 1330 Sofia, Bulgaria; 7 Research Institute, Medical University Pleven, 5800 Pleven, Bulgaria; drstoqn.kostov@gmail.com; 8 Department of Gynecology, Hospital “Saint Anna”, 9002 Varna, Bulgaria

**Keywords:** cervical cancer, HPV screening, Balkans, Eastern Europe, Bulgaria, dual staining, p16/Ki-67, liquid-based cytology, cancer prevention

## Abstract

Cervical cancer is one of the most preventable malignancies, yet disparities in survival remain a significant public health burden across Eastern Europe and the Balkans. Bulgaria illustrates this paradox: HPV vaccination coverage is very low, and prevention continues to rely substantially on opportunistic screening, although recent national initiatives have introduced HPV-based testing and self-sampling. This article argues that the primary barrier to cervical cancer control in Bulgaria is not the lack of medical technology, but the absence of a structured, organized screening system. A modern national cervical cancer screening program should be recognized as a structured clinical and organizational pathway rather than a single diagnostic test. Such a programme requires population identification, active invitation and recall, reg-istry-based tracking, standardized referral and follow-up pathways, and continuous quality assurance. Primary high-risk HPV (hrHPV) testing provides high sensitivity and excellent negative predictive value, supporting longer screening intervals; however, screening for HPV positivity alone is insufficient as a sole referral criterion because many infections are transient and clinically insignificant. We propose a rational framework for cervical cancer screening in Bulgaria and comparable health systems: invitation-based screening for women aged 25–65 years built on primary hrHPV testing with genotyping, reflex cytology, and selective use of p16/Ki-67 dual staining. Drawing on a regional comparison of HPV vaccination and screening programs and on Bulgarian cost-of-illness data, we argue that transitioning from opportunistic testing to an integrated, registry-based screening cascade is essential for effective and equitable cervical cancer prevention in the region.

## 1. Introduction

Cervical cancer is a preventable malignancy and a marker of unequal health-system performance. Its course and biology are now well understood—persistent infection with oncogenic human papillomavirus (HPV) is the necessary causal driver in most cases. Precursor lesions can be detected and treated, and vaccination can prevent infection with most carcinogenic HPV genotypes. Yet the ability to translate these tools into population-level prevention varies substantially between countries [1,2].

The World Health Organization has defined cervical cancer elimination as a global public health priority through the 90–70–90 targets: 90% of girls fully vaccinated against HPV by age 15, 70% of women screened with a high-performance test by ages 35 and 45, and 90% of women with precancer or invasive cervical cancer appropriately treated [3]. These targets are conceptually simple, but achieving them requires organized systems: population registries, active invitations, validated tests, quality assurance, timely diagnostic work-up, treatment capacity, and longitudinal follow-up [2,4].

Eastern Europe and Bulgaria represent a particularly relevant region for this discussion. Countries in the region differ in HPV vaccination coverage, screening organization, public information, registry capacity, and access to specialized gynecological care. This heterogeneity creates a prevention gap: some women are repeatedly screened through private or opportunistic care, while others remain outside the screening pathway entirely. In such settings, the availability of cytology, HPV tests, or colposcopy does not automatically translate into effective cancer prevention [5].

In Bulgaria, cervical cancer remains a significant public health problem, with approximately 23.5 new cases per 100,000 and mortality of 8.4 per 100,000—rates surpassed in the European Union only by Romania and Latvia [6]. These figures are especially concerning because cervical cancer often affects women in active social, professional, and reproductive age groups. The persistence of this burden reflects not only biological risk, but also gaps in prevention organization.

## 2. Current Status in Bulgaria

Cervical cancer screening in Bulgaria has historically been dominated by opportunistic cytology and patient-initiated contact with the healthcare system. Although recent Ministry of Health initiatives have introduced HPV-based testing and self-sampling opportunities, these activities do not yet constitute a fully implemented organized population-based screening programme. At the time of writing, Bulgaria still lacks a nationwide invitation-based system with systematic population registration, recall, standardized triage pathways, and programme-level follow-up. The current landscape should therefore be understood as predominantly opportunistic screening accompanied by emerging national prevention initiatives rather than an established organized screening programme. Women may be screened during routine gynecological visits, private consultations, pregnancy-related care, local campaigns, or patient-initiated examinations. This model can detect disease among women who are already engaged with healthcare services, but it does not ensure equitable coverage of the target population. It also makes it difficult to monitor screening intervals, test quality, follow-up of abnormal results, colposcopy referral, treatment completion, and long-term outcomes.

The consequence is a fragmented prevention landscape. Some women may undergo repeated cytology or HPV testing without a standardized risk-based pathway, while others remain unscreened or under-screened for long periods. Women from rural areas, lower-income groups, marginalized communities, or groups with lower health literacy are particularly vulnerable to being missed by a system that depends on individual initiative rather than active invitation. In this setting, national screening coverage figures may overestimate the effectiveness of prevention if they reflect self-reported testing rather than organized program participation.

HPV vaccination is an essential part of long-term cervical cancer control, but its impact in Bulgaria will depend on sustained uptake, public trust, and integration with screening policy. Reported HPV vaccination coverage remains very low—below 2% among girls aged 9–14 years in 2023 [7]—so for adult women already beyond the vaccinated birth cohorts, screening remains the immediate preventive priority. Therefore, improving cervical cancer prevention in Bulgaria requires more than increasing access to individual tests. It requires transforming fragmented testing into an organized screening cascade with a defined target population, validated primary testing, reflex triage, quality assurance, colposcopy referral thresholds, and reliable follow-up.

Seen in this way, Bulgaria is not only a national case, but also a relevant example of a broader regional challenge. The problem is not the absence of medical technology, but the absence of a coordinated system capable of delivering prevention consistently and equitably.

## 3. The Regional Prevention Gap: Opportunistic Screening Is Not Enough

The distinction between opportunistic and organized screening is clinically meaningful. Opportunistic screening depends on patient initiative, irregular physician visits, local campaigns, or individual access to gynecological care [8]. It may detect disease in women who are already engaged with the health system, but it cannot guarantee equitable population coverage. It also makes quality control, recall, follow-up of positive results, and outcome monitoring difficult.

Organized screening, in contrast, begins with a defined target population and a defined screening interval. Eligible women are actively invited, test results are recorded in a central system, and positive results trigger standardized triage and referral pathways. Colposcopy, histology, treatment, and follow-up are monitored [9]. Program performance can then be evaluated through measurable indicators: screening coverage, HPV positivity, triage positivity, colposcopy referral rate, time to diagnostic confirmation, detection of CIN2+/CIN3+, treatment completion, and invasive cancer incidence.

This distinction is particularly important in the Balkans. In many settings, gynecological care may technically be available, but access is uneven [5]. Urban women, women with higher health literacy, women with private insurance or financial resources, and women already in contact with healthcare services are more likely to be screened [10]. Women from rural areas, marginalized communities, lower-income groups, or those experiencing fear, stigma, or distrust may be missed. A national program must therefore be designed not only to offer screening, but to reach the women least likely to seek it spontaneously.

## 4. Screening Is Not a Test: Why HPV-Positive Women Need Risk-Based Triage

A national HPV screening program should not be reduced to mass HPV testing alone. Without triage and follow-up, a positive screening test does not reliably translate into prevention [1,4,9].

This point is essential for HPV-based screening. A positive high-risk HPV (hrHPV) result indicates infection with an oncogenic HPV type, but it does not necessarily indicate cervical precancer or cancer. Many HPV infections, especially in younger women, are transient and clear without intervention [8]. If all HPV-positive women are referred directly to colposcopy, the program may generate high referral volumes, unnecessary invasive procedures, psychological distress, and increased costs. At the same time, an unclear or delayed pathway after a positive test may fail women with clinically significant disease.

Risk-based triage solves this problem. It retains the sensitivity advantage of primary HPV testing while improving specificity and directing colposcopy toward women with the highest probability of CIN2+, CIN3+, or early invasive disease [9]. The aim is not simply to find HPV, but to identify clinically meaningful HPV-related transformation.

## 5. Comparing Cervical Cancer Screening Methodologies

Several screening methodologies are available, but they differ substantially in performance, resource requirements, and suitability for a new national program (Table 1).

Conventional Pap cytology detects morphologic abnormalities in exfoliated cervical cells. It has historically reduced cervical cancer burden in countries with high repeated coverage and strong cytology quality assurance [11,12]. However, cytology has lower sensitivity than HPV testing and requires frequent repeated participation [9,12]. Its performance depends on sampling quality, slide preparation, cytopathology expertise, and robust recall systems. For countries where screening has remained opportunistic, conventional cytology alone is unlikely to be the optimal foundation for a modern national program.

Liquid-based cytology (LBC) improves the cytological workflow. Cells are collected into a liquid medium, allowing cleaner slides, fewer unsatisfactory samples, and additional testing from the same vial. LBC can improve sample adequacy and laboratory efficiency compared with conventional smears [8,9]. However, LBC should not be considered a replacement for HPV-based screening; its strongest role is as a reflex triage test in women who have already tested positive for hrHPV [13].

Primary hrHPV testing detects the molecular cause of cervical carcinogenesis. It has higher sensitivity for high-grade lesions than cytology and provides strong reassurance after a negative result, allowing longer screening intervals [1,8,9]. However, HPV testing is less specific than cytology because it detects infections that may never progress. Therefore, primary HPV testing requires a structured triage algorithm for positive results.

HPV/cytology co-testing may achieve high detection, but it increases test volume, cost, and downstream follow-up. For a resource-conscious national program—especially one transitioning from opportunistic screening—primary HPV testing with reflex triage is generally more practical than routine co-testing of all women [4,9].

Direct colposcopy for all HPV-positive women is simple but inefficient. It risks overwhelming colposcopy services and exposes many women with transient infection to unnecessary evaluation. This approach may be considered only for clearly defined high-risk situations, such as high-grade cytology or selected HPV16/18-positive pathways, depending on the final national protocol [8].

Self-sampling for HPV testing may improve screening uptake not only among women in rural or underserved areas, but also among those deterred by embarrassment, discomfort, privacy concerns, logistical barriers, or previous negative experiences with gynecological care. It may therefore serve both as an equity tool and as an alternative invitation strategy for women who do not participate in clinician-based screening. Recent real-world experience has shown that integrating HPV self-sampling into screening programmes can increase participation, although positive results still require appropriate clinician-based triage, referral, and follow-up [5,14].

## 6. Proposed Risk-Stratified HPV-Based Screening Framework for Bulgaria and Comparable Balkan Settings

A feasible framework for Bulgaria would comprise organized primary hrHPV screening in women aged 25–65 years, with the starting age and upper age limit periodically reassessed according to national epidemiology, HPV vaccination coverage, programme performance, and emerging European recommendations. This age range should be regarded as a country-specific implementation choice rather than a universally applicable international threshold. The primary screening test should use a clinically validated hrHPV assay capable of identifying HPV16 and HPV18 separately while detecting the remaining high-risk genotypes individually or as a pooled group. Limited HPV genotyping provides an additional level of risk stratification because HPV16/18-positive women have a substantially higher risk of CIN3+ than women positive for other high-risk HPV genotypes [15,16]. Recent WHO guidance specifically recognizes HPV DNA genotyping as a means of stratifying and prioritizing the management of HPV-positive women within screening programmes [16].

Women with a negative primary hrHPV test should return to routine screening after five years. This interval is consistent with contemporary international recommendations and reflects the high negative predictive value of HPV-based screening [1,4,8]. WHO recommends screening intervals of 5–10 years when HPV DNA testing is used as the primary screening test in the general population [17], while the European Council Recommendation supports HPV-based screening at intervals of five years or longer [18]. We therefore selected five years as a conservative initial interval for the proposed Bulgarian framework. Once programme-level outcome data become available, this interval could be reassessed according to cervical cancer incidence, HPV vaccination coverage, screening participation, and longitudinal risk.

Women with a positive hrHPV test should undergo risk-based triage rather than automatic referral to colposcopy. Whenever technically possible, reflex triage from the same clinician-collected specimen is preferable because it limits additional visits and may reduce loss to follow-up. Liquid-based cytology (LBC) can provide morphological risk stratification, while p16/Ki-67 dual-stain cytology may be used selectively when additional discrimination between women requiring colposcopy and those suitable for surveillance is clinically useful [9,19,20]. Dual staining should not, however, be regarded as an obligatory component of every HPV-based screening programme. Rather, it represents one possible biomarker-based triage strategy whose incorporation depends on laboratory capacity, quality assurance, cost, follow-up capacity, and the availability of alternative triage approaches [9].

The optimal triage strategy for hrHPV-positive women remains an area of active clinical debate, and several approaches are currently used or being evaluated. Reflex cytology combined with partial HPV genotyping remains a practical and widely established option, while extended genotyping can provide more refined risk stratification by separating HPV types into different risk categories. Biomarker-based approaches, particularly p16/Ki-67 dual-stain cytology, offer an alternative means of identifying transforming HPV infections and may reduce unnecessary colposcopy referrals, although their implementation requires additional laboratory capacity and quality assurance. Other molecular approaches, including host or viral DNA methylation assays, are also being investigated, but they are not yet uniformly incorporated into routine screening programmes. These strategies differ in sensitivity, specificity, laboratory complexity, cost, and the number of women referred for colposcopy. Therefore, the reflex pathway proposed here should be viewed as one feasible risk-adapted approach for Bulgaria rather than as the only acceptable triage model; the final strategy should reflect validated test performance, available laboratory expertise, colposcopy capacity, and the ability of the programme to ensure reliable follow-up [16,20,21].

Follow-up after an HPV-positive result should be determined by the combination of HPV genotype, cytological or biomarker triage findings, and persistence of infection rather than by HPV positivity alone. The proposed 12- and 24-month surveillance intervals therefore reflect differences in residual risk and should not be interpreted as universal genotype-specific thresholds. Real-world data from the Norwegian cervical screening programme demonstrate substantially higher CIN3+ risk in HPV16/18-positive women than in women positive for other hrHPV genotypes, including among women with normal cytology [15]. Risk-stratified Norwegian screening pathways consequently use earlier reassessment for higher-risk groups. Women with HPV16/18 positivity and NILM cytology have been reassessed at 12 months, whereas women with non-16/18 hrHPV and NILM cytology may undergo repeat HPV testing at 24 months. In contrast, women with non-16/18 hrHPV and low-grade cytology (ASC-US/LSIL) are generally reassessed at approximately 12 months when management is based on cytology and genotyping alone [22].

Dual-stain triage provides an additional basis for extending surveillance in women with a reassuring biomarker result. WHO guidance supports repeat HPV testing at 24 months after a negative p16/Ki-67 dual-stain result in the general population [9]. Accordingly, within the proposed Bulgarian framework, a negative dual-stain result may support a 24-month surveillance interval in appropriately selected HPV-positive women, including women with otherwise low-grade or reassuring triage findings. Where dual staining is not performed and management relies on cytology and genotyping alone, a shorter interval, such as 12 months for non-16/18 hrHPV-positive women with ASC-US/LSIL, may be more appropriate [22]. The precise surveillance interval should therefore be determined by the complete risk profile rather than by HPV genotype in isolation.

**Figure 1:**
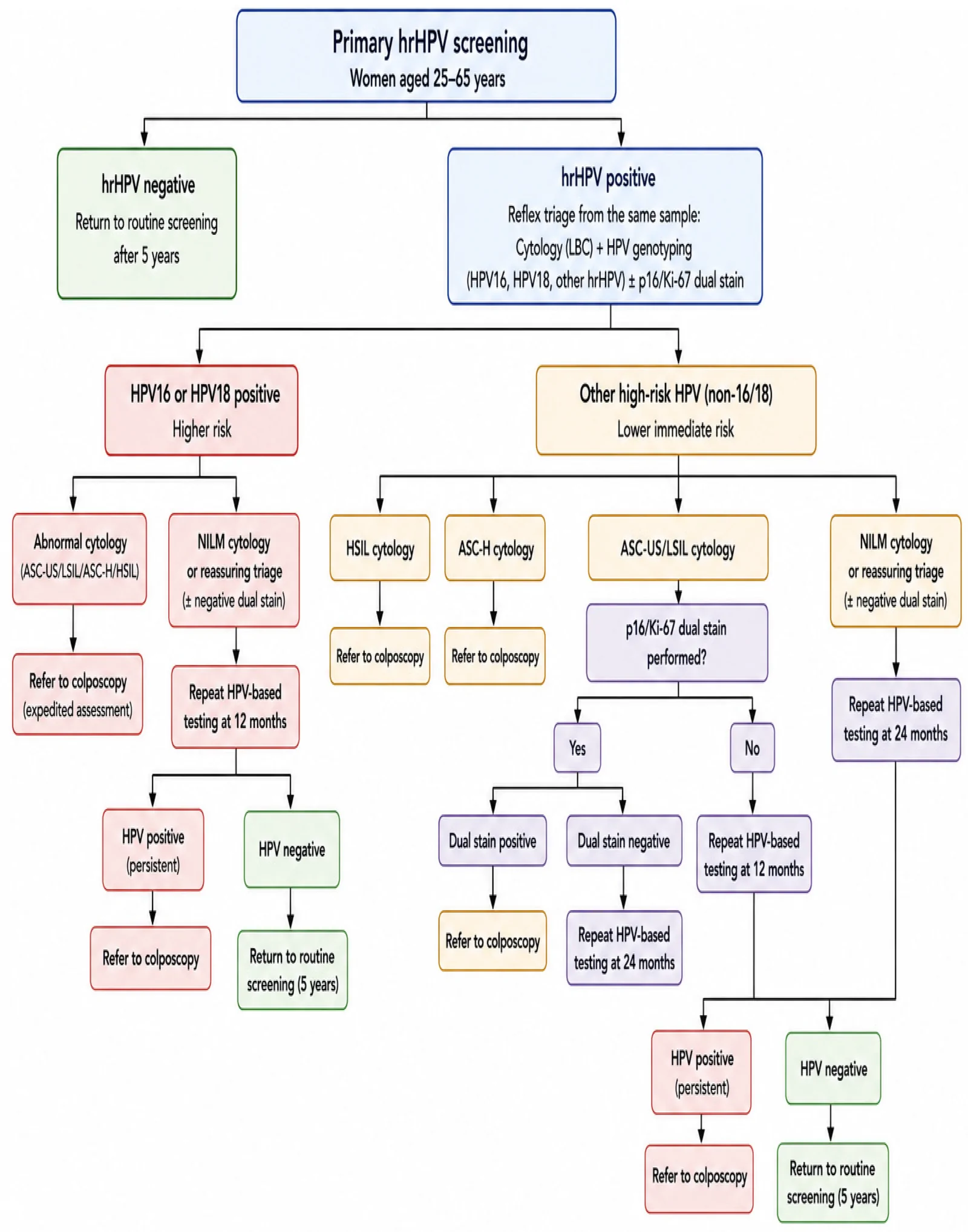
Proposed risk-stratified framework for HPV-based cervical cancer screening in Bulgaria. Abbreviations: hrHPV, high-risk human papillomavirus; LBC, liquid-based cytology; DS, dual stain; NILM, negative for intraepithelial lesion or malignancy; ASC-US, atypical squamous cells of undetermined significance; ASC-H, atypical squamous cells—cannot exclude HSIL; LSIL, low-grade squamous intraepithelial lesion; HSIL, high-grade squamous intraepithelial lesion.

The proposed risk-stratified framework is summarized in Figure 1. Its principal management pathways can be outlined as follows:

- hrHPV negative: Return to routine HPV-based screening after five years.
- hrHPV positive: Perform reflex risk stratification using HPV genotyping and cytology, with selective p16/Ki-67 dual staining where clinically and operationally appropriate.
- HSIL or ASC-H cytology: Refer for colposcopic assessment because of the high immediate risk of clinically significant cervical disease.
- HPV16/18 positivity: Prioritize for expedited assessment because of the higher genotype-specific risk. Women with abnormal cytology should undergo prompt colposcopic evaluation, while women with reassuring cytology or additional triage findings may undergo earlier surveillance according to predefined national risk thresholds; a 12-month repeat HPV test represents one established risk-stratified approach [15,22].
- Non-16/18 hrHPV with ASC-US/LSIL: When management is based on cytology and genotyping, repeat HPV-based testing at approximately 12 months is an established risk-stratified option [22]. Where p16/Ki-67 dual staining is additionally used, dual-stain-positive women should be referred to colposcopy, whereas a negative dual-stain result may support repeat HPV testing at approximately 24 months [9].
- Non-16/18 hrHPV with NILM cytology: Repeat HPV-based testing at approximately 24 months may be appropriate within a risk-stratified pathway [15,22].
- Persistent hrHPV positivity during surveillance: Proceed to further diagnostic evaluation, including colposcopy according to predefined national risk thresholds.
- HPV negativity during surveillance: Return to routine screening.

HPV16 and HPV18 deserve particular consideration because they account for a disproportionate share of cervical cancers and confer substantially greater risk of high-grade precancer than other oncogenic HPV types. In Norwegian real-world data, among HPV-positive women with normal cytology, the observed CIN3+ risks were 19.9% for HPV16, 10.8% for HPV18, and 5.5% for other hrHPV genotypes [15]. These differences support the use of genotype as a risk modifier and justify earlier assessment of HPV16/18-positive women rather than treating all hrHPV-positive results as clinically equivalent. Conversely, women positive for non-16/18 hrHPV constitute a more heterogeneous and generally lower-risk group, in whom cytology, persistence of infection and, where available, biomarker triage can help distinguish women requiring immediate diagnostic evaluation from those suitable for surveillance.

The framework presented here is intended to illustrate how contemporary HPV-based, risk-stratified principles could be adapted to the Bulgarian healthcare setting. It does not represent an established Bulgarian clinical guideline. The five-year primary screening interval is supported by international recommendations [17,18], whereas the precise combination of genotype-specific surveillance, cytology, selective dual-stain triage, and 12- versus 24-month follow-up represents a context-adapted implementation framework informed by existing guidelines and European programme experience. Specific age thresholds, surveillance intervals, triage technologies, and colposcopy referral criteria should therefore be finalized through national consensus and prospectively evaluated after implementation.

## 7. Role of p16/Ki-67 Dual Staining in Risk Stratification

The clinical value of p16/Ki-67 dual stain lies in its ability to distinguish HPV infection from HPV-induced cellular transformation [19]. HPV positivity alone identifies viral exposure or infection. Dual stain identifies abnormal co-expression of p16, a marker associated with HPV-mediated oncogenic activity, and Ki-67, a marker of cellular proliferation, within the same epithelial cell. This combination is biologically meaningful because mature squamous epithelial cells should not normally express both markers together.

In practice, dual stain is particularly useful for HPV-positive women with equivocal, low-grade, or negative cytology—precisely the categories in which direct referral of all women to colposcopy would be inefficient [20]. A dual-stain-positive result supports prompt colposcopy referral, while a dual-stain-negative result can support surveillance with repeat HPV-based testing, provided that recall and follow-up systems are reliable.

Recent clinical evidence has further supported p16/Ki-67 dual staining as a useful triage tool in HPV-positive women. Data from large prospective studies indicate that dual staining can improve risk stratification compared with conventional cytology and may reduce unnecessary colposcopy referrals while maintaining high sensitivity for clinically relevant precancerous lesions [23]. Importantly, dual staining should complement rather than override established high-risk management pathways; women with high-grade cytology or other findings associated with high immediate risk should proceed to appropriate diagnostic assessment regardless of the biomarker result.

When incorporated selectively within an organized screening pathway, dual staining may serve as an additional risk-stratification tool. Its role should depend on available laboratory expertise, quality assurance, cost, and the feasibility of alternative triage strategies. Expert colposcopy capacity is limited, and unnecessary referrals may delay assessment for women at higher risk. Dual stain can help preserve colposcopy resources while maintaining safety, provided it is implemented within a quality-assured LBC-based laboratory system.

## 8. Organizational Requirements and Implementation Challenges

A screening algorithm can succeed only if it is supported by an organized system, and moving from opportunistic testing to an organized HPV-based program requires more than changing the primary screening test. For Bulgaria and comparable Balkan countries, the following elements are essential.

First, a central screening registry is needed to identify eligible women, issue invitations, record results, track follow-up, and monitor outcomes. Without a registry, screening remains a series of disconnected clinical encounters rather than a measurable public health program, and coverage, regional inequalities, time to diagnostic confirmation, detection of CIN2+/CIN3+, and treatment completion cannot be evaluated. A screening registry should be regarded as enabling infrastructure rather than as an intervention that independently improves population-level outcomes. Its principal value is to support identification of the eligible population, invitation and recall, tracking of abnormal results, quality assurance, and monitoring of diagnostic and treatment pathways. Any subsequent reduction in cervical cancer incidence or mortality would depend on the combined performance of the entire prevention pathway, including screening participation, timely triage and colposcopy, treatment completion, adherence to follow-up, HPV vaccination, and equitable access to care.

Second, the program should use active invitations rather than relying on patient initiative. Invitation systems should include mailed letters, electronic notifications, primary-care reminders, and targeted outreach for under-screened groups.

Third, laboratory testing should be centralized or quality-assured. Primary HPV screening requires validated molecular assays, standardized sampling and transport, and quality-assured reporting; where LBC and p16/Ki-67 dual staining are incorporated, laboratories also need appropriate equipment, trained personnel, and external quality control. Centralizing testing may improve quality but must be balanced against timely reporting and equitable regional access.

Fourth, the program must define clear colposcopy referral thresholds. These should include high-grade cytology, dual-stain positivity, HPV16/18 positivity according to agreed risk thresholds, and persistent HPV positivity during follow-up. Because primary HPV testing identifies more positive women than cytology, risk-based triage is necessary not only clinically but also to protect limited colposcopy capacity.

Fifth, screening should be connected to a structured patient pathway with diagnosis, treatment, and outcome monitoring. Detection of CIN2+/CIN3+ is meaningful only if women receive timely treatment and follow-up. Programme performance should initially be evaluated through process and intermediate indicators, including population coverage, timely follow-up of positive results, colposcopy referral, CIN2+/CIN3+ detection, and treatment completion. Reductions in invasive cervical cancer incidence and mortality remain appropriate long-term population-level objectives, but they should be interpreted as outcomes of the integrated prevention pathway rather than of registry-based screening alone.

Sixth, the program should incorporate equity indicators. Coverage should be evaluated by age, region, socioeconomic vulnerability, rural versus urban residence, and participation history. Without these data, national averages may hide persistent disparities.

For Bulgaria, implementation could proceed in phases. The initial priority should be to establish programme governance, define the eligible population, develop a central registry, and ensure access to validated primary HPV testing. A second phase should introduce systematic invitation and recall, standardized referral pathways, adequate colposcopy capacity, and closed-loop follow-up of positive results. Additional strategies, including wider self-sampling, extended genotyping, and p16/Ki-67 dual staining, could then be introduced selectively after pilot evaluation of feasibility, costs, and local capacity.

Beyond these structural requirements, two further challenges are less technical but equally decisive. The first is public trust. HPV testing and HPV vaccination are both linked to a sexually transmitted infection, which can create stigma, misunderstanding, or hesitation. Low confidence in HPV vaccination would weaken long-term prevention, while poor communication around HPV-positive results may increase anxiety and reduce adherence to follow-up. Public information should explain that HPV infection is common, that most infections are transient, and that screening is designed to identify clinically meaningful risk rather than infection alone.

The second is sustainable financing and governance, which will determine whether implementation can move beyond short-term campaigns. Organized screening requires continuous funding for invitations, testing, registry maintenance, laboratory quality assurance, public communication, diagnostic work-up, and treatment. Without long-term coordination between public health authorities, gynecologists, laboratories, primary care, and oncology services, even a well-designed algorithm may remain difficult to translate into practice.

## 9. Economic Rationale for Prevention

Although this perspective does not include a formal cost-effectiveness model, Bulgarian cost-of-illness data provide a strong economic rationale for investing in organized cervical cancer prevention. A national screening program should not be evaluated only by the unit cost of HPV testing, cytology, dual staining, or colposcopy. Its value should be considered in relation to the costs of treating invasive cervical cancer, the loss of productive life years, and the wider societal consequences of preventable disease. However, cost-of-illness evidence should not be interpreted as demonstrating the cost-effectiveness of the specific screening pathway proposed here. While these data quantify the economic burden of cervical cancer and support investment in prevention, the relative costs and health outcomes of different HPV-based screening, triage, and follow-up strategies would require formal health-economic modelling using Bulgarian programme, resource, and epidemiological data.

A recent Bulgarian cost-of-illness study based on National Health Insurance Fund data for 2018–2020 showed that cervical cancer imposes a substantial and increasing economic burden on the healthcare system [24]. Direct healthcare costs rose during the study period, with drug acquisition and administration accounting for the largest share of expenditure, followed by inpatient management. The cumulative direct costs of cervical cancer over 2018–2020 were estimated at EUR 17.68 million, and, because the majority of cases are attributable to HPV, approximately EUR 15.74 million of these costs could potentially be linked to HPV-related disease. Cervical cancer also caused considerable productivity losses, with more than 20,000 years of life lost and more than 5000 working life years lost during the same period [24].

These data are important for screening policy because they show that the economic consequences of cervical cancer occur downstream, after prevention has failed. Treatment of invasive disease requires hospital care, systemic therapy, radiotherapy, surgery, follow-up, and management of complications. By contrast, organized screening aims to detect HPV-related transformation before invasive cancer develops. Even if HPV-based screening requires investment in registries, laboratory capacity, invitations, triage, and colposcopy services, these costs should be weighed against the continuing expenditure associated with late diagnosis and cancer treatment.

The economic argument is also relevant to the choice of screening strategy. Primary HPV testing may have higher upfront laboratory costs than conventional cytology, but it offers higher sensitivity and allows longer screening intervals after a negative result. Its resource implications are strongly influenced by the triage strategy: if all HPV-positive women are referred directly to colposcopy, the program may generate unnecessary costs and overload specialist services, whereas genotyping, reflex cytology, and selective p16/Ki-67 dual staining may help direct colposcopy toward women at higher risk while allowing lower-risk women to undergo surveillance. In this sense, triage is not only a clinical tool, but also a mechanism for rational resource allocation.

For Bulgaria, the key economic issue is therefore not whether prevention requires investment, but how resources can be allocated more effectively across prevention, diagnosis, and treatment. The available national burden data indicate that substantial costs currently accumulate at the treatment stage. A structured HPV-based screening pathway, integrated with vaccination policy and registry-based follow-up, has the potential to shift the focus of resource use toward earlier prevention; however, its cost-effectiveness relative to alternative strategies requires formal evaluation.

## 10. Lessons for the Balkans

The Bulgarian case reflects a broader regional issue. Cervical cancer prevention requires coordinated public health infrastructure in addition to available diagnostic and clinical services. The countries included in the regional comparison were selected as policy-relevant comparators for Bulgaria, covering Balkan and neighbouring Southeast European health systems at different stages of HPV vaccination, screening organization, and registry development. This variation provides an opportunity to identify transferable implementation lessons rather than to rank national programmes. Cross-country comparisons should nevertheless be interpreted cautiously because coverage estimates are not fully harmonized and differ in target populations, reporting years, denominators, and data sources [25,26,27] (Table 2).

HPV vaccination policies differ substantially across the region: some countries have introduced gender-neutral or school-based programs, while others remain limited by low uptake, incomplete catch-up strategies, or inconsistent reporting. However, vaccination policy alone does not explain the regional cervical cancer prevention gap. Screening organization remains equally important, particularly for adult women who are already beyond the vaccinated birth cohorts.

Table 3 compares the organization, target age, primary screening test, and reported coverage of cervical cancer screening programs across the region.

Cross-country coverage estimates should be interpreted with caution because the underlying indicators are not fully harmonized. HPV vaccination data differ in target populations, age groups, dose definitions, reporting years, and denominators, while cervical screening estimates may reflect participation in organized programmes, administrative records, population surveys, IARC/ICO estimates, or regional and subnational data [25,26,27]. Target age ranges and the time periods used to define recent screening also vary. Consequently, the percentages presented in Table 2, Table 3 and Table 4 should not be considered directly comparable measures of national programme performance. Rather, they are intended to illustrate broad differences in policy, organization, and implementation and to identify potentially transferable approaches across the region.

The screening comparison shows that Balkan countries do not follow a single prevention model. Slovenia represents the most mature organized cytology-based program, while Albania, Montenegro, and Türkiye have moved toward HPV-based screening models, and Greece applies age-stratified HPV testing for women aged 30–65 years. Croatia and Bulgaria continue to rely substantially on opportunistic cytology-based screening, despite relatively high self-reported participation. Romania illustrates a different implementation problem: formal program existence with very low program-based coverage. These differences support the central argument that cervical cancer prevention depends not only on the screening test, but also on invitation systems, registry-based follow-up, quality assurance, and clearly defined triage pathways.

To synthesize these overlapping prevention dimensions, Table 4 summarizes the regional prevention gap by combining reported HPV vaccination coverage, cervical cancer screening participation, and age-standardized cervical cancer incidence across selected Balkan countries [5,25,50,51,52].

Slovenia offers a useful benchmark for what organized cervical screening can achieve when invitation systems, registries, quality assurance, and public trust are aligned, as demonstrated by its ZORA program. Romania and Bulgaria illustrate the consequences of delayed or fragmented implementation within the European Union. Serbia, North Macedonia, Albania, Montenegro, Bosnia and Herzegovina, Kosovo, Croatia, Greece, and Türkiye provide important comparators across the wider region, where policy development, financing, vaccination acceptance, and screening access remain heterogeneous.

Three transferable lessons are particularly relevant for Bulgaria. First, the Slovenian experience highlights the importance of a defined target population supported by a central registry, systematic invitation and recall, and continuous quality assurance. Second, programmes in Albania, North Macedonia, and Türkiye illustrate the value of integrating cervical screening with primary care and established community-based health services to improve access and participation. Third, the experiences of countries adopting HPV-based screening, such as Montenegro and Türkiye, suggest that the choice of triage strategy should be matched to available laboratory expertise, follow-up systems, and colposcopy capacity rather than determined by test performance alone. These lessons support a phased Bulgarian model in which organizational infrastructure precedes the introduction of more complex triage technologies.

We believe that opportunistic prevention cannot reliably eliminate cervical cancer. Regional strategies should be adapted to local healthcare structures, but they should share core principles: HPV-based primary screening, risk-based triage, registry-based follow-up, quality assurance, and outreach to under-screened populations.

## 11. Policy Recommendations

Based on established international recommendations, regional experience, and the considerations discussed above, we propose five policy priorities for Bulgaria and the wider Balkan region. These priorities represent a context-adapted implementation framework rather than prescriptive clinical guidelines.

First, transition from opportunistic to organized screening. Screening should be invitation-based, registry-supported, and nationally monitored.

Second, adopt primary hrHPV testing as the backbone of the program. Cytology remains important, but primarily as a triage tool after HPV positivity rather than as the sole screening method.

Third, implement risk-based triage from the start. HPV-positive women should undergo risk-based triage using validated approaches such as HPV genotyping and cytology, with selective p16/Ki-67 dual staining where laboratory capacity and resources permit.

Fourth, define measurable performance indicators. Coverage, HPV positivity, triage positivity, colposcopy referral rate, time to colposcopy, CIN3+ detection, treatment completion, and cancer incidence should be monitored continuously.

Fifth, design screening for equity. Self-sampling pilots, mobile outreach, culturally sensitive education, and primary-care engagement should aim to reach women who do not attend routine gynecological care.

## 12. Limitations

This article is a perspective and does not present original epidemiological analyses, primary survey data, or cost-effectiveness modeling. The proposed framework is based on published evidence, international recommendations, regional comparisons, and expert interpretation, and should therefore be viewed as a policy-oriented proposal rather than a validated national implementation protocol.

The implementation of p16/Ki-67 dual-stain triage may also be challenging in lower-resource settings. Its routine use requires access to validated assays, appropriate laboratory equipment, trained cytotechnologists and pathologists, standardized interpretation, and consistent internal and external quality assurance. Limited laboratory workforce, variable experience with biomarker-based cytology, reagent and equipment costs, and the need to maintain acceptable turnaround times may restrict widespread deployment in some Balkan settings. For this reason, dual staining should be introduced selectively and, where appropriate, through centralized or phased implementation, with conventional cytology and HPV genotyping remaining valid alternative triage strategies where dual-stain capacity is not available [9].

The regional comparisons also have important limitations. Screening coverage estimates are not fully harmonized across countries because they derive from different sources, including program-based administrative data, survey-based self-reporting, IARC/ICO estimates, and regional assessments. Countries also differ in target age groups, screening intervals, registry capacity, test modality, and reporting definitions. HPV vaccination coverage data have similar limitations, particularly where gender-neutral vaccination has only recently been introduced or where implementation varies within a country. For this reason, coverage is presented in categories rather than as exact percentages.

Accordingly, the proposed Bulgarian pathway would require local validation before national adoption. Future work should include pilot implementation, health-economic evaluation, assessment of laboratory and colposcopy capacity, acceptability studies among women and healthcare professionals, and prospective monitoring of program indicators.

## 13. Conclusions

Cervical cancer prevention in Bulgaria and the surrounding Balkan countries should no longer depend on fragmented campaigns or opportunistic cytology. The scientific basis for prevention is established, and successful examples of cervical cancer control have been reported; implementation remains the principal challenge.

A national HPV-based screening programme based on validated hrHPV testing, risk-based triage, organized invitations, registry-based follow-up, and standardized referral pathways would align Bulgaria with contemporary international recommendations. Depending on laboratory capacity, quality assurance, cost, and available resources, triage may incorporate HPV genotyping, reflex liquid-based cytology, and selective p16/Ki-67 dual staining. The purpose of such a programme is not simply to test and diagnose, but to establish a coordinated prevention pathway capable of identifying women at increased risk, avoiding unnecessary procedures, ensuring appropriate follow-up, and ultimately contributing to reductions in cervical cancer incidence and mortality.

In this sense, cervical cancer elimination is both a medical and an organizational responsibility. For Eastern Europe and the Balkans, the next step is not only to know what prevents cervical cancer, but to ensure that prevention reaches every woman.

## Figures and Tables

**Table 1:** Comparison of cervical cancer screening methodologies and their relevance for Bulgaria and the Balkans.

Screening Approach	Main Principle	Strengths	Limitations	Suggested Role in a National Program
Conventional Pap cytology	Morphologic detection of abnormal cervical cells	Familiar, historically effective in high-coverage programs	Lower sensitivity; quality-dependent; requires repeated participation	Not ideal as the foundation of a new program
Liquid-based cytology	Cytology from a preserved liquid sample	Cleaner slides, fewer unsatisfactory samples, possible reflex testing	Still less sensitive than HPV testing as a primary screen	Reflex triage after HPV positivity
Primary hrHPV testing	Molecular detection of high-risk HPV	High sensitivity, high reassurance after a negative result, longer intervals	Lower specificity; detects transient infections	Backbone of organized screening
HPV testing with partial genotyping	Separates HPV16/18 from other high-risk types	Improves immediate risk stratification	Requires validated assays and clear management rules	Essential part of risk-based triage
HPV/cytology co-testing	HPV and cytology performed together	High detection	Higher cost, more follow-up, limited added benefit over HPV alone	Less suitable for a resource-conscious new program
HPV-positive → LBC ± p16/Ki-67	Molecular screening followed by cytology/biomarker triage	Balances sensitivity and specificity; reduces unnecessary colposcopy	Requires LBC and dual-stain capacity	Preferred proposed model
Direct colposcopy for all HPV-positive women	All HPV-positive women referred	Simple	Over-referral, anxiety, cost, service overload	Avoid as a general strategy
Self-sampling HPV testing	Patient-collected sample for HPV testing	Improves access for under-screened groups	Positive results still need clinical triage	Equity tool once registry and recall exist

**Table 2:** HPV vaccination programs and reported vaccination coverage categories in selected Balkan and Southeast European countries.

Country	Program Status	Publicly Funded Eligible Groups	Routine Target Group	Girls’ VCR	Boys’ VCR	Sources
Bulgaria	National publicly funded program expanded to gender-neutral vaccination from 2025.	Girls 10–14; boys 10–13; older girls included through phased catch-up/extension.	Girls 10–14; boys 10–13.	<30%	N/A	[5,28,29]
Romania	National HPV vaccination program with HPV9; reimbursement and eligibility differ by age and sex.	Girls and boys; young-adult reimbursement/catch-up rules apply per national schedule.	Adolescents/young people, per national schedule.	<30%	<30%	[5,28,30]
Croatia	Gender-neutral HPV vaccination is recommended and publicly funded.	Girls and boys; catch-up through young adulthood; 26+ mainly for defined medical-risk groups.	From 5th grade/approx. 11 years.	N/A	N/A	[5,28,29,31]
Slovenia	National routine gender-neutral HPV vaccination program.	Girls and boys in grade 6; latecomer/catch-up available per cohort rules.	Grade 6/approx. 11–12 years.	30–49%	<30%	[5,32]
Serbia	Free HPV vaccination for girls and boys.	Girls and boys aged 9–19.	Adolescents 9–19 years.	<30%	<30%	[5,13]
North Macedonia	National routine HPV vaccination program.	Girls and boys; boys’ coverage not consistently reported in comparative sources.	Adolescent target group.	50–80%	N/A	[5]
Montenegro	National routine gender-neutral HPV vaccination program.	Girls and boys; risk-based and young-adult catch-up per national policy.	Adolescent target group.	<30%	<30%	[5]
Bosnia and Herzegovina	HPV vaccination program reported, but implementation is fragmented.	Girls and boys, with subnational variation.	Adolescent target group.	<30%	N/A	[5]
Albania	HPV vaccination introduced for adolescent girls; program development is ongoing.	Girls; extension to boys pending.	Girls around 13 years.	50–80%	N/A	[5,33]
Kosovo *	National HPV vaccination program reported, with rapid early implementation.	Girls and boys.	Adolescent target group.	>80%	>80%	[5,34]

Abbreviations: VCR, vaccination coverage rate; N/A, not available in the comparative source. Coverage categories follow the HPV Prevention Policy Atlas Europe classification (<30%, 30–49%, 50–80%, >80%, or N/A). Categories are shown rather than exact percentages because countries differ in target cohort, reporting year, denominator, dose schedule, and program maturity. * Kosovo: this designation is without prejudice to positions on status and is in line with UNSCR 1244/1999 and the ICJ Opinion on the Kosovo Declaration of Independence. Sources: compiled from the HPV Prevention Policy Atlas Europe 2025/2026, the ECDC Vaccine Scheduler, WHO Immunization Data, and country-specific public health sources.

**Table 3:** Organization, target age, primary screening test, and coverage of cervical cancer screening programs in Balkan and Southeast European countries.

Country	Organization of Cervical Cancer Screening	Target Age and Interval	Primary Screening Test	Reported Coverage/Participation	Sources
Albania	National program introduced in 2019, delivered through primary health care with free screening and follow-up.	Women 40–50 years; 5-year interval.	High-risk HPV test.	Approx. 40% coverage reported in 2020; more than 45,000 women screened by early 2024.	[35,36]
Bosnia and Herzegovina	Fragmented/subnational approach. Federation of B&H is largely opportunistic, while Republika Srpska has more defined recommendations.	Federation of B&H: no official age range/interval reported in the cited source; Republika Srpska: women 25–60 years, every 3 years.	Cytology/Pap test.	No comparable national coverage estimate. Republika Srpska reported 33% coverage in 2013.	[25]
Bulgaria	Predominantly opportunistic screening. Recent national initiatives include HPV testing and self-sampling, but a fully implemented organized population-based national screening programme is not yet in place.	No effective invitation-based national pathway. Reported EU/OECD coverage is usually presented for women aged 20–69 years.	Cytology/Pap test.	57% of women aged 20–69 reported screening in 2019. Survey-based; not organized-program coverage.	[26]
Croatia	Opportunistic cervical screening remains the dominant model, despite previous attempts to develop organized screening.	Historically cytology-based; no fully functioning organized national program reported in the 2025 country profile.	Cytology/Pap test.	78% self-reported coverage among eligible women in 2019. Survey-based, not program-based administrative coverage.	[37,38]
Greece	National public health prevention program offering age-stratified cervical cancer screening.	Women 21–29 years: Pap if not screened in previous 3 years; women 30–65 years: HPV-DNA if not screened in previous 5 years.	Pap (21–29 y); HPV-DNA (30–65 y).	Comparable program-based coverage not consistently available in the sources reviewed.	[39,40]
Kosovo *	Mixed model, with primary-care invitation in some municipalities and opportunistic screening elsewhere.	Women 21–64 years; 3-year interval.	Cytology/Pap test.	Approx. 2% coverage reported in 2020 in the regional UNFPA assessment.	[25]
Montenegro	HPV-based cervical screening program with primary testing in health centers/gynecological services.	Women 30–64 years; 5-year interval.	HPV DNA test, with cytology triage after a positive result.	27% screened in the previous 3 years and 38% in the previous 5 years (2019 IARC/ICO estimates).	[27,41]
North Macedonia	National program with primary-care involvement and central screening registry reported in the regional assessment; HPV primary screening piloted.	Women 22–60 years; 3-year interval.	Cytology/Pap test.	Approx. 22% coverage reported in 2017.	[25]
Romania	National program initiated in 2012, with HPV-DNA pilot implementation in selected regions.	National Pap-based: women 25–64 years, every 5 years; HPV-DNA pilot: women 30–64 years.	Cytology/Pap nationally; HPV-DNA in pilot settings.	Program-based coverage very low in 2023, approx. 6–6.2% among women aged 20–69 years.	[42,43]
Serbia	Organized program exists, but implementation remains uneven and partly opportunistic.	Women 25–64 years; Pap smear every 3 years.	Cytology/Pap test.	IARC/ICO estimate 37% screened within previous 3 years (women 25–65); other survey estimates differ by indicator and time frame.	[44,45]
Slovenia	Mature organized population-based program, ZORA, with central registry and invitation/recall mechanisms.	Women 20–64 years; conventional cytology every 3 years after two negative entry tests.	Conventional cytology/Pap, with HPV triage for selected abnormalities.	Approx. 71% 3-year coverage and 86% 5-year coverage in the target population.	[46]
Türkiye	National population-based program delivered through primary care and cancer screening centers.	Women 30–65 years; 5-year interval.	HPV-DNA test, with cytology/Pap in the pathway.	Estimates vary: regional UNFPA reported 44% (2020); WHO country profile reported 82% of women aged 30–49 screened within previous 5 years.	[25,47,48]

* Kosovo: designation as defined in the note to Table 2. Sources: compiled from national program documents, OECD/European Commission country cancer profiles, IARC/ICO and CanScreen5 data, WHO country profiles, UNFPA regional assessments, and peer-reviewed literature (see reference—[49]).

**Table 4:** Descriptive comparison of HPV vaccination coverage, cervical screening participation, and cervical cancer incidence across selected Balkan countries. Darker shading indicates a larger prevention gap or higher disease burden.

Country	HPV Vaccination Coverage	Cervical Screening Coverage/Participation	Cervical Cancer Incidence
**Romania**	**<30%** high gap	**6.2%** high gap	**21.7 ASR** high burden
**Montenegro**	**<30%** high gap	**27%** high gap	**12.0 ASR** moderate burden
**Bulgaria**	**<30%** high gap	**57%** mild gap	**23.5 ASR** high burden
**Serbia**	**<30%** high gap	**37%** moderate gap	**13.4 ASR** moderate burden
**Bosnia and Herzegovina**	**<30%** high gap	33% * moderate gap	**11.2 ASR** moderate burden
**North Macedonia**	**50–80%** mild gap	**22%** high gap	**6.7 ASR** low burden
**Albania**	**50–80%** mild gap	**40%** moderate gap	**8.7 ASR** low burden
**Kosovo ‡**	**>80%** low gap	**2%** high gap	**N/A** not available
**Slovenia**	**30–49%** moderate gap	**71%** low gap	**7.2 ASR** low burden
**Croatia**	**N/A** not available	**78% †** low gap	**8.3 ASR** low burden

Color scale: green, low gap/burden; yellow, mild; orange, moderate; red, high; grey, not available. ASR, age-standardized incidence rate per 100,000 women, World standard population; incidence estimates refer to GLOBOCAN 2022. * Bosnia and Herzegovina: the screening value refers to Republika Srpska in the cited regional source, not harmonized national coverage. † Croatia: self-reported (survey-based) screening coverage, not program-based administrative coverage. ‡ Kosovo: designation as defined in the note to Table 2. Sources: HPV vaccination coverage categories were derived from the European HPV Prevention Policy Atlas 2025 [5]. Cervical screening participation estimates were obtained from OECD country health-system reviews and UNFPA regional assessments [25,50,51], together with the country-specific sources cited in Table 3. Cervical cancer incidence represents the estimated age-standardized incidence rate per 100,000 women, standardized to the world population, from GLOBOCAN 2022 [52]. Screening estimates are not fully harmonized and may represent programme-based coverage, survey-reported participation, or regional/subnational data. Because source definitions, denominators, target populations, reporting periods, and data-collection methods differ across countries, the gap categories shown in Table 4 are descriptive and should not be interpreted as a formal ranking of national programme performance.

## Data Availability

No new data were created or analyzed in this study. Data sharing is not applicable to this article.

## References

[1] Bouvard V., Wentzensen N., Mackie A., Berkhof J., Brotherton J., Giorgi-Rossi P., Kupets R., Smith R., Arrossi S., Bendahhou K. (2021). The IARC perspective on cervical cancer screening. N. Engl. J. Med..

[2] World Health Organization Cervical Cancer Fact Sheet.

[3] World Health Organization Cervical Cancer Elimination Initiative.

[4] European Commission Initiative on Cervical Cancer European Guidelines on Cervical Cancer Screening and Diagnosis. https://cancer-screening-and-care.jrc.ec.europa.eu/en/ec-cvc/european-cervical-cancer-guidelines.

[5] European Parliamentary Forum for Sexual and Reproductive Rights (2025). European HPV Prevention Policy Atlas, 2025/2026 ed..

[6] Todorova I., Panayotova Y., Kotzeva T., Greenley R., McKee M., CBIG-SCREEN Consortium (2026). Cervical cancer in Bulgaria since EU accession in 2007: A struggle in the face of political instability. Health Policy.

[7] European Cancer Organisation Bulgaria Expands National HPV Vaccination. https://www.europeancancer.org/resources/news/bulgaria-expands-national-hpv-vaccination.

[8] International Agency for Research on Cancer (2022). Cervical Cancer Screening.

[9] World Health Organization (2024). WHO Guideline for Screening and Treatment of Cervical Pre-Cancer Lesions for Cervical Cancer Prevention: Use of Dual-Stain Cytology to Triage Women After a Positive Test for Human Papillomavirus (HPV).

[10] Gakidou E., Nordhagen S., Obermeyer Z. (2008). Coverage of cervical cancer screening in 57 countries: Low average levels and large inequalities. PLoS Med..

[11] Hashmi A.A., Naz S., Ahmed O., Yaqeen S.R., Irfan M., Asif M.G., Kamal A., Faridi N. (2020). Comparison of liquid-based cytology and conventional Papanicolaou smear for cervical cancer screening: An experience from Pakistan. Cureus.

[12] Maheshwari Y., Handa U., Aggarwal P., Goel B. (2023). Comparative analysis of conventional cytology and liquid-based cytology in the detection of carcinoma cervix and its precursor lesions. J. Cytol..

[13] Andersen B., Njor S.H., Jensen A.M.S., Johansen T., Jeppesen U., Svanholm H. (2020). hrHPV testing vs liquid-based cytology in cervical cancer screening among women aged 50 and older: A prospective study. Int. J. Gynecol. Cancer.

[14] Suwantaroj J., Sripan P., Kallawicha K., Tso J., Singweratham N., Siewchaisakul P. (2025). The Utilization of Human Papillomavirus Testing for Cervical Cancer Screening in Thailand: A Comparison of Screening with and without Self-sampling Samples. Asian Pac. J. Cancer Prev..

[15] Hashim D., Engesæter B., Baadstrand Skare G., Castle P.E., Bjørge T., Tropé A., Nygård M. (2020). Real-world data on cervical cancer risk stratification by cytology and HPV genotype to inform the management of HPV-positive women in routine cervical screening. Br. J. Cancer.

[16] World Health Organization (2026). WHO Guideline for Screening and Treatment of Cervical Pre-Cancer Lesions for Cervical Cancer Prevention: Use of Human Papillomavirus (HPV) DNA Genotyping.

[17] World Health Organization (2021). WHO Guideline for Screening and Treatment of Cervical Pre-Cancer Lesions for Cervical Cancer Prevention.

[18] Council of the European Union (2022). Council Recommendation of 9 December 2022 on strengthening prevention through early detection: A new EU approach on cancer screening replacing Council Recommendation 2003/878/EC. Off. J. Eur. Union.

[19] Clarke M.A., Wentzensen N., Perkins R.B., Garcia F., Arrindell D., Chelmow D., Cheung L.C., Darragh T.M., Egemen D., Guido R. (2024). Recommendations for use of p16/Ki-67 dual stain for management of individuals testing positive for human papillomavirus. J. Low. Genit. Tract Dis..

[20] Harper D.M., Paczos T., Ridder R., Huh W.K. (2025). p16/Ki-67 dual stain triage of individuals positive for HPV to detect cervical precancerous lesions. Int. J. Cancer.

[21] Thrall M.J., McCarthy E., Mito J.K., Rao J., Clinical Practice Committee of the American Society of Cytopathology (2025). Triage options for positive high-risk HPV results from HPV-based cervical cancer screening: A review of the poten-tial alternatives to Papanicolaou test cytology. J. Am. Soc. Cytopathol..

[22] Bjørge T., Støer N.C., Hverven S.K., Nygård M., Tropé A., Engesæter B. (2025). Risk of high-grade cervical lesions in the second round of primary human papillomavirus testing in CervicalScreen Norway: A population-based cohort study. Int. J. Cancer.

[23] Zouzoulas D., Chatzistamatiou K., Tsolakidis D. (2025). The role of p16/Ki-67 dual staining in cervical cancer screening and cervical precancerous lesions follow-up. CytoJournal.

[24] Lebanova H., Stoev S., Naseva E., Getova V., Wang W., Sabale U., Petrova E. (2023). Economic Burden of Cervical Cancer in Bulgaria. Int. J. Environ. Res. Public Health.

[25] United Nations Population Fund (UNFPA) Eastern Europe and Central Asia (2021). Situation Analysis of Capacities for Cervical Cancer Prevention, Treatment and Care in Eastern Europe and Central Asia.

[26] OECD/European Commission (2025). EU Country Cancer Profile: Bulgaria 2025.

[27] ICO/IARC Information Centre on HPV and Cancer Montenegro: Human Papillomavirus and Related Cancers, Fact Sheet 2023. https://hpvcentre.net/statistics/reports/MNE_FS.pdf.

[28] European Centre for Disease Prevention and Control ECDC Vaccine Scheduler: Human Papillomavirus Infection. https://vaccine-schedule.ecdc.europa.eu/.

[29] Bulgaria Expands National HPV Vaccination Programme. Ecancer News.

[30] World Health Organization Immunization Data: Human Papillomavirus (HPV) Vaccination Coverage. https://immunizationdata.who.int/.

[31] European Centre for Disease Prevention and Control ECDC Vaccine Scheduler: Croatia (Human Papillomavirus). https://vaccine-schedule.ecdc.europa.eu/Scheduler/ByDisease?SelectedDiseaseId=38.

[32] National Institute of Public Health (NIJZ), Slovenia HPV Vaccination Coverage Indicator (K3.5). https://obcine.nijz.si/en/kazalniki/K3.5.

[33] Pojani E., Bozo S., Hoxha B. (2025). Knowledge, Attitudes and Practices Towards HPV Vaccination among Albanian Women: An Effort to Improve HPV Vaccine Acceptance. Open Public Health J..

[34] European Cancer Organisation (2026). Vaccination Delivery. Good Practice Guide on HPV Vaccination.

[35] Institute of Public Health, Albania Albania National Cervical Cancer Screening Program: Evaluation Report, 2020. https://www.ishp.gov.al/wp-content/uploads/2021/06/2report-evaluation-screening-program-final-2020.pdf.

[36] World Health Organization Regional Office for Europe Albania Upgrades Its Cervical Cancer Screening Programme with WHO’s Support. https://www.who.int/europe/news/item/17-11-2020-albania-upgrades-its-cervical-cancer-screening-programme-with-who-s-support.

[37] OECD/European Commission (2025). EU Country Cancer Profile: Croatia 2025.

[38] OECD/European Commission Croatia’s Cancer Performance Tracker (CaPTr). https://www.oecd.org/.

[39] Hellenic Republic National Program for the Prevention of Cervical Cancer. https://www.ehealthrecord.gov.gr/en/health-actions/prolipsi-canc-trachilos.

[40] Palmer C., Skroumpelos A., Sabale U., Gountas I., Trimis G., Karokis A., Agorastos T. (2025). Strategies to accelerate cervical cancer elimination in Greece: A modeling study. Front. Oncol..

[41] Csanádi M., Filipi K., Ylli A., Dedja B., Bejko A., Nikcevic Kovacevic I., Vukovic-Lekovic J., Stanisic M., Vujovic A., Obeng G.D. (2025). Barriers of organized cervical cancer screening in Albania and Montenegro. BMC Public Health.

[42] Eurostat Cancer Screening Statistics. https://ec.europa.eu/eurostat/statistics-explained/index.php?title=Cancer_screening_statistics.

[43] International Agency for Research on Cancer CanScreen5: Cervical Cancer Screening Programme—Romania. https://canscreen5.iarc.fr/.

[44] Screening Serbia Cervical Cancer Screening. https://www.skriningsrbija.rs/eng/cervical-cancer-screening/.

[45] Institute for Health Economics Cervical Cancer: Improving Care and Driving Policy Change—Serbia. https://ihe.se/app/uploads/2026/01/IHE_POLICY-BRIEF_Cervical-Cancer_Serbia_.pdf.

[46] ZORA Slovenian National Cervical Cancer Screening Programme. https://zora.onko-i.si/en/.

[47] Arslan H.N., Oruc M.A. (2022). Results from a cervical cancer screening program in Samsun, Turkey. BMC Womens Health.

[48] World Health Organization Cervical Cancer Country Profile: Türkiye. https://cdn.who.int/media/docs/default-source/country-profiles/cervical-cancer/cervical-cancer-tur-2021-country-profile-en.pdf.

[49] United Nations Population Fund (UNFPA) Stepping Up HPV Vaccinations for Adolescents to Prevent Cervical Cancer in Serbia. https://eeca.unfpa.org/en/news/stepping-hpv-vaccinations-adolescents-prevent-cervical-cancer-serbia.

[50] OECD (2026). OECD Reviews of Health Systems: Bulgaria 2026.

[51] OECD (2025). OECD Reviews of Health Systems: Romania 2025.

[52] Ferlay J., Ervik M., Lam F., Laversanne M., Colombet M., Mery L., Piñeros M., Znaor A., Soerjomataram I., Bray F. (2024). Global Cancer Observatory: Cancer Today.

